# Profile of Environmental Chemicals in the Korean Population—Results of the Korean National Environmental Health Survey (KoNEHS) Cycle 3, 2015–2017

**DOI:** 10.3390/ijerph19020626

**Published:** 2022-01-06

**Authors:** Sun Kyoung Jung, Wookhee Choi, Sung Yeon Kim, Sooyeon Hong, Hye Li Jeon, Youngkyung Joo, Chulwoo Lee, Kyungho Choi, Sungkyoon Kim, Kee-Jae Lee, Jiyoung Yoo

**Affiliations:** 1 Environmental Health Research Division, National Institute of Environmental Research, Ministry of Environment, Incheon 22689, Korea; sk0825@korea.kr (S.K.J.); harok2@korea.kr (S.Y.K.); suyeon83@korea.kr (S.H.); hyeli0922@korea.kr (H.L.J.); jooyk02@korea.kr (Y.J.); leecwoo@korea.kr (C.L.); 2 Graduate School of Urban Public Health, University of Seoul, Seoul 02504, Korea; 3 Monitoring and Analysis Division, Wonju Regional Environmental Office, Ministry of Environment, Wonju 26461, Korea; whc405@korea.kr; 4 Graduate School of Public Health, Seoul National University, Seoul 08826, Korea; kyungho@snu.ac.kr (K.C.); ddram2@snu.ac.kr (S.K.); 5 Department of Information Statistics and Data Science, College of Natural Science, Korea National Open University, Seoul 03087, Korea; kjlee@mail.knou.ac.kr

**Keywords:** children and adolescents, environmental chemicals, KoNEHS, national biomonitoring, temporal trend

## Abstract

The Korean National Environmental Health Survey (KoNEHS) program provides useful information on chemical exposure, serves as the basis for environmental health policies, and suggests appropriate measures to protect public health. Initiated on a three-year cycle in 2009, it reports the concentrations of major environmental chemicals among the representative Korean population. KoNEHS Cycle 3 introduced children and adolescents into the analysis, where the blood and urine samples of 6167 participants were measured for major metals, phthalates, phenolics, and other organic compounds. Lead, mercury, cadmium, metabolites of DEHP and DnBP, and 3-phenoxybenzoic acid levels of the Korean adult population tended to decrease compared to previous survey cycles but remained higher than those observed in the US or Canada. Both bisphenol A (BPA) and *trans,trans*-muconic acid concentrations have increased over time. Heavy metal concentrations (blood lead, and cadmium) in children and adolescents were approximately half that of adults, while some organic substances (e.g., phthalates and BPA) were high. BPA showed higher levels than in the US or Canada, whereas BPF and BPS showed lower detection rates in this cycle; however, as these are increasingly used as a substitute for BPA, further research is necessary. As environmental chemicals may affect childhood health and development, additional analyses should assess exposure sources and routes through continuous observations.

## 1. Introduction

Human biomonitoring is an important tool for assessing internal exposure to environmental chemicals. Its history extends back to the 1890s, when exposure levels among occupational workers were measured in the workplace [1]. Since then, different studies have reported exposure to numerous substances through various pathways, outlining the utility of biomonitoring in environmental health management [2,3,4,5]. Indeed, several countries, including the United States, Canada, and Germany, have implemented national biomonitoring programs (NBPs) to inform the development of environmental health policies [4,6,7]. NBPs can generate exposure profiles of major environmental chemicals among representative populations, providing valuable information for prioritizing chemicals and policies [3,6,8,9,10].

The Korean National Environmental Health Survey (KoNEHS) has been conducted in three-year cycles since its implementation in 2009. Three cycles have thus been conducted up to 2017, providing baseline information on major chemical exposure profiles. Cycles 1 and 2 were focused on the adult population (n = 12,789), measuring a total of 16 and 21 chemicals, respectively, including metals, bisphenol A (BPA), and metabolites of phthalates [11,12]. 

KoNEHS Cycle 3 (2015–2017) extended the population coverage to include children and adolescents. Many studies have reported that environmental chemicals are related to delayed growth and development in children and adolescents, who are particularly vulnerable to environmental pollution and related chronic diseases [11,13,14,15,16]. Accordingly, this importance was reflected in designing the KoNEHS Cycle 3, building upon the results of the Korean Environmental Exposure and Health Survey in Children and Adolescents (KorEHS-C) from 2012 to 2014 [17,18,19]. A total of 26 environmental chemicals were measured, including substances that became social issues such as parabens [20,21] and bisphenol substitutes [22,23,24]. 

This study provides the updated exposure profiles for major environmental chemicals among the representative Korean population, thus helping to prioritize chemicals of concern that warrant further environmental health management efforts.

## 2. Materials and Methods

### 2.1. Survey Design and Procedures

To ensure representativeness of the adult population, the results of the 2010 Population and Housing Census (Statistics Korea) were used, and a stratified multi-stage sampling process was conducted. Through a household visit in the sampled survey district, participation was asked, and the survey was conducted with those who consented.

These sampling and field survey methods were the same as in Cycles 1 and 2 [11,12], the details of which can be found in Table 1. 

Considering the participant accessibility and ease of sample collection, children and adolescents were extracted in units of kindergartens, childcare facilities, and educational institutions. Using households as sampling units enabled comprehensive sampling across a range of populations; however, children and adolescents who did not attend childcare or educational institutions may be omitted. However, the proportion of that those who attending such institutions was higher than >90%, which would be representative of the same age groups in Koreans [25].

Sampling institutions and sample sizes by age were selected based on the status of daycare centers and educational institutions published by the Ministry of Education and the Ministry of Health and Welfare The country was divided into five regions to be used as variables: Seoul, Gyeonggi/Incheon/Gangwon, Chungcheong, Honam/Jeju, and Yeongnam. In total, 127 schools and 56 kindergartens/childcare facilities were selected across all regions following the sampling process for the KorEHS-C [25].

This survey was approved by the Institutional Review Board of the National Institute of Environmental Research (NIER), Korea (NIER-2015-BR-006-01) and carried out only for those providing prior consent.

### 2.2. Biological Sample Collection

Through a field survey, 18 mL of blood and 60 mL of spot urine were collected from 6167 participants. We sent a message to each subjects notifying them of relevant information 2 days before the survey began. On the day of a field survey, we guided them to the location by mobile phone and finally requested their participation. For adults, samples were taken at the survey site. For preschoolers and children, urine samples were directly collected in a specimen cup at their homes on the day of survey. For adolescents, urine samples were similarly collected upon visiting a pre-negotiated hospital near the investigation. 

In adults, 26 substances in their blood and urine were analyzed (Table 2), whereas in the cases of preschoolers and children, blood sample collections were difficult due to safety considerations, so 25 substances in urine (excepting lead) were analyzed. Mercury is a substance that is analyzed in both whole blood and urine, and the results of mercury in the urine of preschool children and children can be confirmed in Table 3. 

### 2.3. Questionnaire

Questionnaire items were continuous with Cycles 1 and 2, focusing on demographic and socioeconomic characteristics, living environments, recent diet, and lifestyle to identify exposure factors and routes of environmental chemicals. 

A single household member representing preschoolers and elementary school students completed both the household and individual questionnaire surveys. Completed questionnaires were submitted to kindergartens, childcare facilities, and schools to be collected later. For middle and high school students, one household member representing a student completed the common household questionnaire, and individual questions were conducted face-to-face by a trained interviewer. For adults, survey questionnaires were completed solely through face-to-face interviews.

### 2.4. Environmental Chemical Analysis and Quality Assurance/Control 

The biological samples collected in the field survey were transported under cold conditions (2–6 °C), with temperatures checked using a temperature logger (TR52-S temperature data logger, ThermoFisher). The samples were aliquoted from the center within 24 h, and kept frozen at −20 °C until analysis. All processes were performed according to the “Biological sample management guidelines of the KoNEHS Cycle 3 [26]”.

Of the 26 target chemicals selected (Table 2), nine were added compared to the KoNEHS Cycle 2, including endocrine disrupting chemicals and substances expected to increase exposure due to increased usage: mono(3-carboxypropyl) phthalate (MCPP), mono-(carboxynonyl) phthalate (MCNP), mono-(carboxyoctyl) phthalate (MCOP), bisphenol F, bisphenol S, methyl-paraben, ethyl-paraben, propyl-paraben, and N-Acetyl-S-(benzyl)-L-cysteine. 

The target chemicals were analyzed according to the “Analysis manual of the KoNEHS Cycle 3” [27,28], and the equipment used for each chemical analysis are listed in Table 2. The analytical laboratories participated in external quality control programs (e.g., G-EQUAS in Germany, and proficiency testing by NIER) twice a year, and periodic quality assurance/quality control (e.g., linearity and slope of the calibration curve, detection limit, accuracy, and precision) measurements were performed. Table S1 summarizes the analytical methods and limits of detection (LOD) for target chemicals.

### 2.5. Statistical Analyses

Measured values were log-transformed and analyzed using the survey means procedure, after accounting for stratification variables and survey sample weight. Measurements below the LOD were assigned a value equal to LOD/√2 for the calculation of geometric means [6,29]. When non-detects were ≥40%, the arithmetic and geometric means were not calculated. Statistical significance was confirmed with a P value of less than 0.05. SAS software (9.4; SAS Institute Inc., Cary, NC, USA) was used for all statistical analyses.

## 3. Results

### 3.1. Participant Characteristics

The study population consisted of preschoolers (3–5 years, N = 571), children (6–11 years, N = 887), adolescents (12–18 years, N = 922), and adults (≥19 years; N = 3787), for a total of participants of 6167 (2815 males and 3352 females). Within the adults, the proportion of older subjects > 50 years was high, especially higher number of females present among participants >50 years as well. Many more female adult contacts than young people or elderly male for academic or work reasons were made when visiting households for recruitment. Furthermore, the voluntary participation rate was higher for women than men. 

Children and adolescents were recruited through educational institutions; the gender ratios of the final participants were maintained by adjusting for age and gender when recruiting. 

### 3.2. Metals

The geometric mean (GM) concentration of blood lead in adolescents (0.802 μg·dL^−1^) were approximately half those of adults (1.60 μg·dL^−1^; Table 3), as were those of blood mercury (1.37 μg·L^−1^ and 2.75 μg·L^−1^, respectively; Table 3). The GM concentrations of urinary mercury in preschoolers, children, adolescents, and adults were 0.422 μg·L^−1^, 0.394 μg·L^−1^, 0.413 μg·L^−1^, and 0.355 μg·L^−1^, respectively (Table 3); whereas those of urinary cadmium were 0.108 μg·L^−1^, 0.232 μg·L^−1^, 0.289 μg·L^−1^ and 0.359 μg·L^−1^, respectively (Table 3).

### 3.3. Phthalates Metabolites, BPA, and Triclosan (TCS)

The GM concentration in preschoolers, children, and adolescents were mono(2-ethyl-5-hydroxyhexyl) phthalate (MEHHP): 34.6 μg·L^−1^, 28.8 μg·L^−1^, and 13.6 μg·L^−1^; Mono(2-ethyl-5-oxohexyl) phthalate (MEOHP): 25.5 μg·L^−1^, 19.2 μg·L^−1^, and 9.24 μg·L^−1^; and Mono(2-ethyl-5-hydroxyhexyl) phthalate (MECPP): 45.3 μg·L^−1^, 44.5 μg·L^−1^, and 28.4 μg·L^−1^, respectively. Accordingly, phthalate metabolite concentrations in preschoolers were approximately 2–3 times higher than those of adults: MEHHP, 13.2 μg·L^−1^; MEOHP, 9.88 μg·L^−1^; MECPP, 23.2 μg·L^−1^. For MCNP, MCOP, and MCPP added in Cycle 3, the GM concentrations were similar in preschoolers, children, adolescents, and adults: MCNP: 0.491 μg·L^−1^, 0.533 μg·L^−1^, 0.452 μg·L^−1^, and 0.441 μg·L^−1^; MCOP: 1.62 μg·L^−1^, 2.24 μg·L^−1^, 1.71 μg·L^−1^, and 1.07 μg·L^−1^; and MCPP: 1.80 μg·L^−1^, 1.56 μg·L^−1^, 1.48 μg·L^−1^, and 1.13 μg·L^−1^, respectively (Table 3). 

The GM concentrations of urinary BPA decreased with age: 2.41 μg·L^−1^, 1.70 μg·L^−1^, 1.39 μg·L^−1^, and 1.18 μg·L^−1^ in preschoolers, children, adolescents, and adults, respectively (Table 3). The GM concentrations of urinary TCS in preschoolers, children, and adolescents were 0.513 μg·L^−1^, 0.452 μg·L^−1^, and 0.420 μg·L^−1^, respectively (Table 3). 

### 3.4. Parabens

The GM concentrations of methyl- and propyl parabens were relatively high in preschoolers (46.3 μg·L^−1^ and 4.36 μg·L^−1^, respectively). Notably, the 95th percentile of urinary methyl paraben in preschoolers was 3445 μg·L^−1^, ≤10 times higher than those of other age groups. The preschooler concentrations of propyl paraben showed a similar pattern, with a 95th percentile of 699 μg·L^−1^, ≤6 times greater than other age group populations. The GM concentrations of urinary ethyl paraben in preschoolers, children, and adolescents were 14.2 μg·L^−1^, 11.4 μg·L^−1^, 19.1 μg·L^−1^, respectively (Table 3). 

### 3.5. 3-PBA and Cotinine 

Children maintained slightly higher GM concentrations of urinary 3-phenoxybenzoic acid (3-PBA): 1.08 μg·L^−1^, 1.36 μg·L^−1^, 1.02 μg·L^−1^, and 0.965 μg·L^−1^ (Table 3) for preschoolers, children, adolescents, and adults, respectively. 

The GM concentrations of urinary cotinine in adolescents were significantly higher than for preschoolers and children: 1.05 μg·L^−1^, 1.20 μg·L^−1^, 3.04 μg·L^−1^, and 5.59 μg·L^−1^ for preschoolers, children, adolescents, and adults, respectively (Table 3). For adults, the cotinine concentrations of smokers (524 μg·L^−1^) was significantly higher than that of non-smokers (1.87 μg·L^−1^). 

### 3.6. Polycyclic Aromatic Hydrocarbon (PAH) and Volatile Organic Compound (VOC) Metabolites

The GM concentration of urinary 1-hydroxypyrene (1-OH-Pyr) in children, adolescents, and adults was 0.108 μg·L^−1^, 0.163 μg·L^−1^, and 0.130 μg·L^−1^ (Table 3). The concentrations of urinary 2-hydroxynaphthalene (2-NAP) in preschoolers, children, adolescents, and adults were 3.37 μg·L^−1^, 2.67 μg·L^−1^, 3.05 μg·L^−1^, and 2.63 μg·L^−1^, respectively, notably higher for preschoolers (Table 3). The GM concentrations of urinary 1-hydroxyphenanthrene (1-OH-Phe) in preschoolers, adolescents, and adults were 0.080 μg·L^−1^, 0.127 μg·L^−1^ and 0.117 μg·L^−1^, respectively; whereas those of urinary 2-hydroxyfluorene (2-OH-Flu) were notably higher for preschoolers compared to children, adolescents, and adults: 0.495 μg·L^−1^, 0.209 μg·L^−1^, 0.263 μg·L^−1^, and 0.321 μg·L^−1^, respectively.

The GM concentrations of urinary *trans,trans*-muconic acid (t,t-MA) in preschoolers, children, adolescents, and adults were 82.2 μg·L^−1^, 91.2 μg·L^−1^, 80.4 μg·L^−1^, and 86.2 μg·L^−1^, respectively; whereas those of urinary N-Acetyl-S-(benzyl)-L-cysteine (BMA) were 10.6 μg·L^−1^, 7.39 μg·L^−1^, 5.66 μg·L^−1^, and 4.63 μg·L^−1^, respectively.

## 4. Discussion

### 4.1. Comparisons with Other National Biomonitoring Programs 

Comparisons were made between the results of KoNEHS Cycle 3 (2015–2017), the United States National Health and Nutrition Examination Survey (NHANES, 2015–2016), and the Canadian Health Measures Survey (CHMS, 2016–2017; Table S2). 

In preschoolers, BPA (2.41 μg·L^−1^) and paraben (methyl paraben: 46.3 μg·L^−1^ and propyl paraben: 4.36 μg·L^−1^) GM concentrations were 3–4 times higher than in Canada (BPA: 0.94 μg·L^−1^, methyl paraben: 9.9 μg·L^−1^ and propyl paraben: 1.2 μg·L^−1^). In children, paraben (methyl paraben: 28.9 μg·L^−1^ and propyl paraben: 1.83 μg·L^−1^) GM concentrations were 2–3 times higher than in Canada (methyl paraben: 7.5 μg·L^−1^ and propyl paraben: 0.96 μg·L^−1^). Methyl paraben GM concentrations were similar to that of children in the US (methyl paraben: 28.9 μg·L^−1^), but two times lower in adolescents (KoNEHS: 26.1 μg·L^−1^ and US: 40.5 μg·L^−1^) and adults (KoNEHS: 35.2 μg·L^−1^ and US: 52.2 μg·L^−1^). 

In children and adolescents, cadmium (0.232 μg·L^−1^ and 0.289 μg·L^−1^) concentrations were four times higher than in the US (0.057 μg·L^−1^ and 0.055 μg·L^−1^), where levels were highest in the Asian ethnic group compared to others [30]. For adolescents, mercury (1.37 μg·L^−1^) GM concentrations were four times higher than in the US (0.395 μg·L^−1^).

The DEHP metabolite and MnBP, 3-PBA GM concentrations were 2–3 times higher in all age than in the US and Canada. The concentrations are presented in detailed in Table S2. In particular, 3-PBA (GM 95% CI: 1.21–1.52) in Korea was higher than those of other Asian countries, including China (GM 95% CI: 0.08–0.97) and Japan (GM 95% CI: 0.33–1.16) [31]. In adults, GM concentrations of heavy metals (Pb: 1.60 μg·dL^−1^, Hg: 2.75 μg·L^−1^ and Cd: 0.359 μg·L^−1^) were 2–3 times higher than in the US (Pb: 0.920 μg·dL^−1^, Hg: 0.810 μg·L^−1^ and Cd: 0.174 μg·L^−1^) and Canada (Hg: 0.72 μg·L^−1^ and Cd: 0.22 μg·L^−1^). Moreover, DEHP metabolites, and MnBP (MEHHP: 13.2 μg·L^−1^, MEOHP: 9.88 μg·L^−1^, MECPP: 23.2 μg·L^−1^ and MnBP: 22.3 μg·L^−1^) were 2–3 times higher than in the US (MEHHP: 5.29 μg·L^−1^, MEOHP: 3.29 μg·L^−1^, MECPP: 8.12 μg·L^−1^ and MnBP: 9.18 μg·L^−1^) and Canada (MEHHP: 4.7 μg·L^−1^, MEOHP: 3.1 μg·L^−1^, MECPP: 5.5 μg·L^−1^ and MnBP: 11 μg·L^−1^).

### 4.2. Metals

Blood lead concentrations in adults were approximately twice those observed in adolescents, similar to the findings of other research [4,32]. Both drinking and smoking were associated with higher lead concentrations [33,34,35]. Further, it was found here that lead concentrations were higher in adults who had smoked or consumed alcohol more than three times a week (*p* < 0.001). A previous study showed that it has been shown that alcohol consumption in adults accounted for the largest proportion of changes in lead concentrations, followed by smoking [36]. Additionally, infants born to woman who smoke and drink were found to be at greater risk for lead toxicity [37]. Blood lead levels tended to decrease in all countries over time, with a notable larger decline in Korea. Korea implemented a phasing out policy on leaded gasoline from January 1, 1993, making the supply of unleaded gasoline compulsory since then. The rapid decrease in atmospheric lead concentrations, and subsequent gradual decrease in blood lead concentrations was judged to be the primary driver of the observed patterns in Korea [38]. 

In adolescents, blood mercury concentrations were half those of adults. Overall mercury levels were positively associated with fish, egg, meat, and vegetable intake, similar to findings that fish are a potential cause of total mercury exposure among Swedish adolescents [39]. Other studies have shown that even despite low fish consumption, they remained correlated to adolescent blood mercury concentrations [40]. Among the adults of Cycle 3, those living in the coastal regions (GM 3.34 μg·L^−1^) showed higher blood mercury levels than those in urban (GM 2.76 μg·L^−1^) or rural areas (GM 2.64 μg·L^−1^; data not shown). We confirmed similar results in previous studies. It has been demonstrated that very high blood mercury levels detected in Yeongnam region of Korea were attributable to a local culture of consuming shark meat [41]. Analyzing the mercury concentrations, based on the dietary food intake records of 553 adults revealed that fish and shellfish contributed most to the mercury concentration, accounting for 77.8% of the total intake. The results showed that high exposure levels of blood mercury in Korean adults were related to the frequency of fish and shellfish intake [42]. Similar results were reported in the US NHANES data [30,43]. 

The concentration of urinary cadmium increased with age, similar to the findings from other studies [44,45]. The primary exposure source of cadmium among the general population is food, which is related to diet [46,47,48], with the consumption of rice (and other cereals), a notable staple of the East Asian diet, suggested as the major cause of urinary cadmium [49,50]. Rice consumption of the Korean general population is higher than other Asian countries, possibly further contributing to their high cadmium exposure levels. In the present study, grain (non-adults, *p* = 0.004) and soybean (non-adults, *p* < 0.001; adults, *p* < 0.001) intake increased with age, with a further positive correlation revealed between GM cadmium concentrations and soybean intake (non-adults, *p* < 0.001; adults, *p* < 0.001). Monitoring cadmium exposure in the Iranian population indicated that cadmium contamination occurred in food groups, such as rice, cereals, legumes, and vegetables. Specifically, 75% of the consumed rice samples showed cadmium concentrations higher than the maximum cadmium limit (0.06 mg⸱kg^−1^) allowed by the Institute of Standards and Industrial Research of Iran (ISIRI) [51]. In addition, canned fish (mean: 0.032 μg·g^−1^) and tuna (mean: 0.022 μg·g^−1^) samples showed high cadmium concentrations (ISIRI: 0.05 mg⸱kg^−1^) [52,53]. Therefore, a more detailed analysis of food types, consumption frequency, and consumption patterns is required.

### 4.3. Phthalates Metabolites, BPA, and TCS

The concentrations of phthalate, BPA, and TCS in this analysis were higher in preschoolers and children. Children’s ratio of body area to body weight is higher than that of adults, so any correlated exposure to harmful substances through the skin is higher [54,55].

Several studies have similarly shown that the younger the age, the higher the concentration of phthalates in one’s body [56]. Estimating phthalate concentrations in 129 Danish children and adolescents (6–21 years) showed that phthalates were detectable in almost all samples, that youngest children were generally more exposed to phthalates than older children and adolescents [57]. The results of a Portuguese study showed that a healthy diet consisting of fresh, unprocessed or less packaged foods can significantly reduce phthalate intake in children [58]. It was also found that phthalate intakes may be lower in children on a healthy diet (*p* < 0.05) than on a common diet. Several findings have confirmed that childhood phthalate exposure is associated with obesity [59,60,61], which indicates the need for continued management of phthalate exposure in growing children. Although phthalates can cause endocrine disorders, they are widely used in cosmetics, toys, detergents, and household flooring; thus, the mouthing behavior of infants and toddlers could potentially increase their exposure from toys and other products made with plasticized polymers [55]. The younger the children, the greater the frequency of consuming boiled water from plastic containers (*p* < 0.001), and the correlated MEHHP and MEOHP GM concentrations were significantly high (*p* = 0.005, *p* = 0.019, respectively).

Higher BPA levels in younger ages have also been observed in several other studies [62,63,64]. BPA is commonly used in various products for everyday use, including water-pipes, electronic equipment, paper, or toys [65]. A Greek child cohort study showed that BPA was associated with exposure to plastics and personal care products. In addition, the risk of neurotoxic activity makes BPA more likely to affect children’s health more readily [66]. For children, the amount of percutaneous chemical absorption is three times that of adults, with particular exposure risks to harmful substances adsorbed on flooring, or other indoor products [67,68]. Food is the most important source of BPA exposure in the general population [7]. Particularly, canned foods have been shown to maintain significantly higher rates of BPA exposure, as it is released from lacquer coatings on the tins [69,70]. Although exposure sources, such as canned beverages and canned foods, were analyzed here, no significant correlations with BPA concentrations were observed, whereas liquids and other consumer products in polycarbonate containers have previously been shown to increase urinary BPA [71].

When investigating the association between TCS and the use of personal care products, the former was detected in >70% of children, with notable higher concentrations in the group using hand soap, increasing with the frequency of hand washing. Additionally, the use of toothpaste by children was also positively related to TCS, further explaining its high correlation with personal care products [72]. In the present study, higher frequency use of antibacterial products (*p* = 0.021) lead to higher concentrations of TCS in non-adults, whereas adult concentrations were significantly correlated to frequency of body wash use (*p* = 0.041).

### 4.4. Parabens

In the case of methyl and propyl paraben (commonly used together), concentrations in preschoolers were relatively higher. Parabens are often used as preservatives in medicines, such as pills and liquid antipyretics [73], both of which are commonly consumed by preschoolers [74,75,76,77]. Generally higher urinary methyl and propyl paraben concentrations among adult females can be explained by the more frequent use of personal care products among females, as both are commonly used as preservatives in such items [78]. A previous study of paraben concentrations in the urine of the Belgian population also showed that paraben exposure patterns differ significantly between children and adults, and between men and women [79]. The levels of methyl (33.5 μg·L^−1^, *p* < 0.001) and propyl (3.3 μg·L^−1^, *p* < 0.001) parabens were higher in women, highlighting their association with cosmetic and personal care product use. For both methyl and propyl paraben, males were higher in infancy, whereas females were far higher in adolescence and adulthood. Methyl paraben is one of the most frequently used preservatives in cosmetic products such as lipsticks, perfumes, and personal care products [80], thus potentially explaining the higher levels found in girls [81]. Ethyl paraben showed the highest concentrations in adults, where urinary levels were positively associated with the consumption of fast and canned foods. Use of liquid soaps, including shower gel and shampoo, was also associated with ethyl paraben levels [77]. An analysis of ethyl paraben intake by food type according to the result of the Korea National Health and Nutrition Examination Survey, found it was primarily consumed through sauces and mixed soy sauce [82]. Therefore, further detailed analyses are needed on food types, ingredients, and current consumption patterns. 

### 4.5. 3-PBA and Cotinine

The GM concentrations of urinary 3-PBA in children was slightly higher than that of other age groups. Further, children < 9 years had greater 3-PBA concentrations when living in rural residential apartments compared to similar urban locations, indicating the effects of landscaping pesticides on nearby playgrounds and public areas [83]. Various studies have found that sprays and fumigant-type insecticides were also important contributors to 3-PBA exposure of the Korean population [7,84,85]. In adults, 3-PBA concentration were higher when more time was spent at home (*p* = 0.0006) than outdoors, and coffee intake was also found to have an effect (*p* = 0.007) [86]. Further research is needed regarding exposure from consuming beverages and foods, as well as the amount of time spent indoors and outdoors.

Adolescent males showed significantly higher cotinine concentrations than females. As age increased, the number of male adolescents who started smoking increased while the number of female adolescents decreased [87]. According to the KoNEHS Cycle 3 survey results, the smoking rate was 3.1% for adolescents (63% male, 31% female, 6% do not know), and 15.9% for adults (90% male, 10% female). As the frequency of secondhand smoke increased, so too did cotinine concentrations [88], indicating the potential indirect effects of smoking on children and adolescents. It was confirmed that cotinine concentrations in non-adults were higher if there was a smoker in the family (*p* < 0.001), or when more time was spent exposed to secondhand smoke (*p* < 0.001). Adults also showed higher cotinine concentrations with family smokers present (*p* < 0.001), and secondhand smoke exposure (*p* = 0.001).

### 4.6. PAH and VOC Metabolites

The GM concentrations of urinary 2-NAP and 2-OH-Flu in preschoolers were significantly higher than the other age ranges. When food was cooked using gas, 2-NAP concentrations were significantly higher in non-adults (*p* = 0.005), whereas increased frequencies of charcoal grilling was correlated with higher 1-OH-Phe concentrations in non-adults (*p* = 0.032). PAHs metabolites were detected in over 78% of urine samples from 522 children, aged 5–12 years of Valencia, Spain, and 2-NAP was detection rate in 100%. In addition to the consumption of legumes and packaged foods, the education level, demographic characteristics, socioeconomic characteristics were previously identified as the most relevant factors influencing PAHs exposure levels in children [89]. Children were more susceptible to PAHs exposure than adults [90], and higher concentrations of PAH metabolites were found when household cooking was conducted with heating fuels and gases [91]. The concentrations of 1-OH-Pyr, 2-NAP, 1-OH-Phe, and 2-OH-Flu in adults were all significantly higher when petroleum was used as the household heating fuel (*p* = 0.001, *p* < 0.001, *p* < 0.001, and *p* = 0.001, respectively). Elsewhere, studies have indicated that diet, smoking, and air pollution were major determinants of internal PAHs exposure [91,92,93]. In addition to one’s smoking status, the consumption of certain foods (such as vegetables, oils and fats, smoked fish and coffee) has been shown to be a major factor influencing PAHs exposure [94]. Further research is needed on the exposure to different types of heating fuels, food intake and cooking methods.

The t,t-MA concentrations were higher in men than women, across all ages (Table S3), and exposure levels were significantly higher in smokers than non-smokers [95,96]. The higher smoking rate among men is thought to drive these patterns. Additionally, food consumption containing sorbic acid may be another potential source t,t-MA. Sorbic acid is used as a preservative, and ~0.05–0.5% is metabolized to t,t-MA after ingestion [97]. 

The GM concentrations of urinary BMA in preschoolers were 10.6 μg·L^−1^, significantly higher than either children or adolescents (7.39 μg·L^−1^, and 5.66 μg·L^−1^, respectively). According to the results of the National Health and Nutrition Survey in the US, the BMA concentrations in children were higher than in adults. This can be explained either by higher childhood levels of exposure to chemicals, such as ethylbenzene-styrene and toluene, or slower excretion rates than that of adults [98]. A previous study of urban childcare facilities found the detected VOC metabolites was correlated to the use of chlorine bleach and scented candles; thus, higher concentrations could be related to the exposure of children spending greater amounts of time in childcare facilities compared to home [99]. 

## 5. Conclusions

The KoNEHS is the largest biomonitoring survey capable of identifying environmental chemical exposure levels across a nationally representative population. The most current exposure profiles for major environmental chemicals among the Korean population, KoNEHS Cycle 3 (2015–2017), is summarized here. 

In Korean children and adolescents, concentrations of mercury and cadmium were higher than in other countries. Although these levels are not of great concern when compared with Human Biomonitoring Commission recommendations, continuous observations are required to ensure they are not deleteriously affecting children’s growth, development, or health. 

BPA and paraben levels were also high; further studies are needed due to the increased use of bisphenol substitutes (bisphenol F, S) and parabens, and there were different trends according to gender and age. In addition, the levels of phthalate biomarkers measured in the KoNEHS were higher in children and adolescents than in adults. Accordingly, the results indicated the need for exposure reduction measures throughout basic lifestyle and daily life, while continuously monitoring children’s exposure in particular. Among Korean adults, lead, mercury, cadmium, metabolites of DEHP and DnBP, and 3-PBA levels were higher than those reported in the US or Canada; however, their concentrations have been decreasing with time. Urinary BPA and t,t-MA concentrations showed an increasing trend, both warranting further studies on their exposure pathways. 

For children and adolescents, the Cycle 3 survey represented the first national biomonitoring results. Although it was not possible to explain the exact mechanism of substances with a large concentration difference compared to foreign standards, it is believed that the effect will be due to differences in dietary habits and the resulting basic body shape and living environment. Additional research is needed on the effects of exposure to environmental chemical substances. Moving forward, if data are secured through continuous surveys, it will be possible to identify various exposure factors by comparing data over time and analyzing connections within the survey results. Furthermore, The Ministry of Environment of Korea has established a “concentration standard in the human body” and is conducting a detailed investigation by reviewing whether these are exceeded through the results of the KoNEHS. Findings from these efforts will be used to develop environmental health policies, and appropriate mitigation measures for protecting the health of the people.

## Figures and Tables

**Table 1:** Survey sample design details of the KoNEHS Cycle 3 (2015–2017).

	Method
Survey design	Cross-sectional survey
Target population	Over 3 years (2015–2017) living in the Korea Age ≥ 3, male/female
Sampling unit	Housing Census (2010) List of nationwide kindergartens and daycares (2014) List of elementary, middle, and high schools nationwide Apartment and general enumeration district households
Target error	±5% of the average value for each item
Sample size	A minimum sample of ≥5500 participants Infants ≥ 3 years; ~500 (56 institutions) Elementary, middle, and high school students; ~1500 (127 schools) Adults ≥ 19 years; ~3500 (233 survey districts)
Sampling frame · stratifications · characteristics · classification indicators (dwelling and participant)	[Preschoolers] Multi-stage stratified cluster sampling Sampling of collection sites (5 areas), Regions (city or town) stratifications 1st sampling unit: Institutes (kindergarten and daycares) : Age, sex group strata, random sampling 2nd sampling unit: Individual [School age children] Multi-stage stratified cluster sampling Sampling of collection sites (5 areas), Regions (city or town), and school stratifications 1st sampling unit: Institutes (schools) : Age, sex group strata, random sampling 2nd sampling unit: Individual (class and students) [Adults] Multi-stage stratified cluster sampling 17 regions (city or town) stratifications 1st sampling unit: Sampling district 2nd sampling unit: Household
Sample allocation methods	For region (city or town) stratifications: Square root proportional distribution method For detailed stratifications: Proportional allocation method Apply relative standard error after analysis

**Table 2:** Target chemicals of urinary analyses measured in KoNEHS Cycle 3 (2015–2017).

Category	Chemicals	Analytical Technique
Metals (3)	Lead ^a^	GF-AAS,
		(Analyst 800, PerkinElmer, Waltham, MA, USA)
	Mercury (total) ^a^	DMA ^b^
		(DMA-80, Milestone, Shelton, CT, USA)
	Cadmium	GF-AAS,
		(240Z, Agilent, Santa Clara, CA, USA)
Phthalates metabolites (8)	Mono-(2-ethyl-5-oxohexyl) phthalate (MEOHP)	UPLC-MS/MS (LCMS-8060, Shimadzu, Kyoto, Japan)
	Mono-(2-ethyl-5-hydroxyhexyl) phthalate (MEHHP)	
	Mono-(2-ethyl-5-carboxypentyl) phthalate (MECPP)	
	Mono-n-butyl phthalate (MnBP)	
	Monobenzyl phthalate (MBzP)	
	Mono(3-carboxypropyl) phthalate (MCPP) *	
	Mono-(carboxynonyl) phthalate (MCNP) *	
	Mono-(carboxyoctyl) phthalate (MCOP) *	
Environmental phenols (7)	Bisphenol A	UPLC-MS/MS (LCMS-8060, Shimadzu, Kyoto, Japan)
	Bisphenol F *	
	Bisphenol S *	
	Triclosan	
	Ethyl paraben *	
	Methyl paraben *	
	Propyl paraben *	
Pyrethroid pesticides metabolite (1)	3-phenoxybenzoic acid	GC-MS
		(Clarus 600T, Perkin Elmer, Waltham, MA, USA)
Tobacco smoke metabolite (1)	Cotinine	GC-MS
		(Clarus 680-SQ 8T, PerkinElmer, Waltham, MA, USA)
PAHs metabolites (4)	1-Hydroxypyrene (1-OH-Pyr)	GC-MS (7890A/5975C, Agilent, Santa Clara, CA, USA)
	2-Hydroxynaphthalene (2-NAP)	
	1-Hydroxyphenanthrene (1-OH-Phe)	
	2-Hydroxyfluorene (2-OH-Flu)	
VOC metabolites (2)	*trans,trans*-muconic acid	HPLC-MS/MS
	N-Acetyl-S-(benzyl)-L-cysteine *	(Agilent 6410B/1200, Agilent, Santa Clara, CA, USA)

^a^ Specimens: Whole blood; ^b^ Direct mercury analyzer; * Substances added to Cycle 3.

**Table 3:** Distribution of concentrations of environmental chemicals for the KoNEHS Cycle 3 (2015–2017). Units for all chemicals are in μg·L^−1^, except for lead, which is expressed as μg·dL^−1^.

	Age	N	%<LOD	AM	GM	(95% CI ^a^)	P25	P50	P75	P95
Whole Blood										
Metals										
Lead (ug·dL^−1^)	12–18	912	2.74	0.883	0.802	(0.754, 0.853)	0.611	0.834	1.10	1.52
	≥19	3747	0.1	1.79	1.60	(1.56, 1.65)	1.20	1.60	2.20	3.36
Mercury (total)	12–18	912	0	1.56	1.37	(1.30, 1.43)	1.00	1.35	1.84	3.02
	≥19	3745	0	3.50	2.75	(2.63, 2.88)	1.75	2.71	4.15	8.81
Spot Urine										
Metals										
Mercury (total)	3–5	571	2.10	0.565	0.422	(0.371, 0.479)	0.257	0.396	0.693	1.30
	6–11	887	1.24	0.581	0.394	(0.370, 0.418)	0.267	0.373	0.562	1.09
	12–18	906	3.31	0.577	0.413	(0.374, 0.457)	0.246	0.421	0.680	1.39
	≥19	3780	6.1	0.518	0.355	(0.335, 0.376)	0.196	0.339	0.616	1.42
Cadmium	3–5	571	18.7	0.160	0.108	(0.086, 0.136)	0.057	0.091	0.206	0.430
	6–11	887	1.92	0.298	0.232	(0.212, 0.255)	0.146	0.231	0.354	0.735
	12–18	906	1.99	0.377	0.289	(0.264, 0.316)	0.182	0.297	0.445	0.951
	≥19	3781	6.8	0.615	0.359	(0.326, 0.395)	0.185	0.422	0.811	1.75
Phthalates metabolites										
MEHHP	3–5	571	0	45.9	34.6	(31.3, 38.2)	21.3	36.2	58.7	96.8
	6–11	885	0	37.5	28.8	(26.5, 31.2)	18.7	29.6	47.2	85.3
	12–18	901	0.11	19.9	13.6	(12.0, 15.5)	8.39	14.8	24.6	53.6
	≥19	3781	0.4	23.1	13.2	(12.0, 14.4)	6.89	13.7	25.9	62.6
MEOHP	3–5	571	0	34.3	25.5	(23.0, 28.3)	15.2	26.2	40.9	83.6
	6–11	885	0	25.7	19.2	(17.6, 20.9)	11.8	19.5	31.6	65.3
	12–18	901	0.22	14.1	9.24	(8.02, 10.7)	5.10	10.3	17.1	38.5
	≥19	3781	0.5	18.1	9.88	(8.89, 11.0)	5.03	10.7	20.8	50.9
MECPP	3–5	571	0	65.9	45.3	(38.4, 53.5)	25.6	46.7	80.9	173
	6–11	885	0	59.0	44.5	(40.9, 48.5)	27.2	44.0	72.6	144
	12–18	901	0	36.8	28.4	(25.3, 31.8)	18.0	28.5	45.7	89.7
	≥19	3781	0	40.3	23.2	(20.8, 25.7)	11.4	21.7	44.0	131
MnBP	3–5	571	0.53	79.8	47.2	(40.8, 54.6)	32.2	52.9	79.1	157
	6–11	885	0	55.9	43.2	(40.6, 46.1)	28.3	45.1	68.8	126
	12–18	897	0.11	60.6	36.9	(29.6, 46.0)	18.6	36.5	76.8	168
	≥19	3779	1.1	45.6	22.3	(19.2, 25.8)	12.5	25.3	52.0	125
MBzP	3–5	571	3.68	8.06	3.12	(2.65, 3.69)	1.62	3.21	6.83	22.0
	6–11	885	5.99	6.85	2.80	(2.34, 3.35)	1.35	3.17	7.02	24.2
	12–18	901	3.77	6.91	2.78	(2.36, 3.27)	1.19	2.97	6.90	25.3
	≥19	3781	2.0	4.40	1.99	(1.80, 2.20)	0.932	2.05	4.30	14.6
MCNP	3–5	571	4.90	0.670	0.491	(0.425, 0.568)	0.316	0.464	0.685	1.83
	6–11	885	2.26	0.661	0.533	(0.505, 0.562)	0.408	0.526	0.674	1.45
	12–18	901	3.11	0.535	0.452	(0.423, 0.483)	0.331	0.499	0.594	1.02
	≥19	3781	10.5	0.613	0.441	(0.395, 0.493)	0.228	0.502	0.774	1.47
MCOP	3–5	571	0	2.27	1.62	(1.43, 1.83)	0.894	1.55	2.66	6.76
	6–11	885	0.11	3.38	2.24	(2.07, 2.42)	1.28	2.13	3.79	8.31
	12–18	901	1.89	2.41	1.71	(1.55, 1.88)	1.07	1.73	2.94	6.83
	≥19	3781	0.6	1.74	1.07	(0.968, 1.19)	0.569	1.06	1.95	4.59
MCPP	3–5	571	0.18	2.28	1.80	(1.63, 1.99)	1.25	1.70	2.57	5.30
	6–11	885	0.45	1.97	1.56	(1.49, 1.64)	1.07	1.45	2.08	4.43
	12–18	901	0.22	1.75	1.48	(1.35, 1.62)	1.03	1.38	2.09	3.89
	≥19	3781	0.6	1.63	1.13	(1.02, 1.25)	0.672	0.997	2.02	3.84
Environmental phenols										
Bisphenol A	3–5	571	1.23	4.33	2.41	(2.05, 2.83)	1.43	2.60	4.39	10.6
	6–11	887	3.95	3.22	1.70	(1.49, 1.95)	0.849	1.98	3.59	10.1
	12–18	904	3.10	2.65	1.39	(1.20, 1.61)	0.755	1.45	2.84	9.13
	≥19	3780	2.1	2.51	1.18	(1.06, 1.32)	0.517	1.32	2.76	7.78
Bisphenol F	3–5	571	69.9	*	*	*	<LOD	<LOD	0.093	0.761
	6–11	884	65.3	*	*	*	<LOD	<LOD	0.132	1.20
	12–18	900	56.0	*	*	*	<LOD	<LOD	0.153	1.48
	≥19	3777	59.3	*	*	*	<LOD	<LOD	0.141	1.23
Bisphenol S	3–5	571	47.5	*	*	*	<LOD	0.020	0.046	0.186
	6–11	884	46.0	*	*	*	<LOD	0.023	0.065	0.523
	12–18	900	38.2	0.293	0.053	(0.041, 0.068)	<LOD	0.036	0.120	1.08
	≥19	3776	45.7	*	*	*	<LOD	0.022	0.057	0.288
Triclosan	3–5	571	21.9	1.57	0.513	(0.435, 0.606)	0.208	0.400	1.02	4.79
	6–11	887	32.6	3.52	0.452	(0.404, 0.507)	<LOD	0.316	0.828	6.05
	12–18	904	35.3	3.96	0.420	(0.369, 0.479)	<LOD	0.315	0.714	4.75
	≥19	3780	50.9	*	*	*	<LOD	<LOD	0.538	5.39
Methyl Paraben	3–5	571	0	646	46.3	(37.1, 57.8)	8.81	25.8	150	3445
	6–11	884	0	188	28.9	(24.6, 33.9)	7.16	18.8	91.3	913
	12–18	900	0	107	26.1	(21.9, 31.2)	7.17	18.2	91.5	510
	≥19	3779	0	128	35.2	(32.0, 38.6)	9.26	34.6	126	506
Ethyl Paraben	3–5	571	1.05	106	14.2	(10.5, 19.1)	2.66	17.2	66.2	526
	6–11	884	1.13	122	11.4	(8.56, 15.3)	1.96	10.6	69.0	504
	12–18	900	0.44	85.6	19.1	(14.0, 26.2)	4.88	19.0	71.6	350
	≥19	3779	1.0	158	30.9	(27.1, 35.2)	7.82	36.2	139	676
Propyl Paraben	3–5	571	0.70	153	4.36	(3.29, 5.80)	0.664	2.48	18.0	699
	6–11	884	5.43	30.7	1.83	(1.52, 2.21)	0.346	1.37	7.15	104
	12–18	900	2.11	38.7	3.19	(2.55, 3.98)	0.796	2.11	10.8	212
	≥19	3778	3.9	51.0	3.07	(2.73, 3.46)	0.464	2.12	16.9	224
Pyrethroid pesticides metabolites										
3-PBA	3–5	570	0.35	2.24	1.08	(0.870, 1.33)	0.536	0.970	1.86	9.34
	6–11	887	0.68	2.40	1.36	(1.21, 1.52)	0.749	1.29	2.39	7.44
	12–18	904	1.66	2.03	1.02	(0.842, 1.24)	0.565	1.12	2.18	5.93
	≥19	3772	0.9	1.82	0.965	(0.887, 1.05)	0.499	1.03	2.05	6.09
Environmental tobacco smoke metabolites										
Cotinine	3–5	571	20.5	1.69	1.05	(0.913, 1.20)	0.450	1.19	2.29	4.80
	6–11	887	18.9	2.12	1.20	(1.05, 1.38)	0.521	1.48	2.82	5.84
	12–18	904	7.19	36.1	3.04	(2.50, 3.70)	1.40	2.79	4.72	85.2
	≥19	3784	6.7	240	5.59	(4.80, 6.50)	0.799	1.80	12.7	1530
19 years and older	None	3181	84.0	39.6	1.87	(1.65, 2.12)	0.698	1.30	3.10	75.6
	Smoker	603	16.0	1071	524	(393, 700)	429	1008	1504	2575
PAHs metabolites										
1-OH-Pyr	3–5	554	47.5	*	*	*	<LOD	0.080	0.353	1.02
	6–11	864	34.4	0.319	0.108	(0.091, 0.127)	<LOD	0.208	0.442	1.02
	12–18	867	31.5	0.613	0.163	(0.115, 0.230)	<LOD	0.338	0.804	2.21
	≥19	3754	28.4	0.364	0.130	(0.118, 0.143)	<LOD	0.201	0.434	1.28
2-NAP	3–5	554	1.26	6.30	3.37	(2.89, 3.92)	1.64	3.31	7.16	21.3
	6–11	864	3.82	5.63	2.67	(2.37, 3.01)	1.45	2.71	5.16	19.8
	12–18	866	2.77	6.29	3.05	(2.63, 3.53)	1.69	3.15	6.40	20.2
	≥19	3754	1.3	5.59	2.63	(2.48, 2.79)	1.23	2.42	6.16	21.0
1-OH-Phe	3–5	554	38.3	0.156	0.080	(0.069, 0.093)	<LOD	0.054	0.194	0.499
	6–11	864	41.8	*	*	*	<LOD	0.168	0.360	0.933
	12–18	866	38.2	0.265	0.127	(0.107, 0.151)	<LOD	0.158	0.367	0.822
	≥19	3751	32.5	0.269	0.117	(0.109, 0.126)	<LOD	0.135	0.303	0.779
2-OH-Flu	3–5	549	19.7	2.18	0.495	(0.327, 0.750)	0.133	0.589	1.98	10.7
	6–11	864	20.0	0.380	0.209	(0.178, 0.246)	0.085	0.279	0.513	1.08
	12–18	867	15.9	0.485	0.263	(0.225, 0.307)	0.145	0.317	0.647	1.40
	≥19	3754	14.1	0.697	0.321	(0.295, 0.349)	0.159	0.379	0.780	2.58
VOCs metabolites										
t,t-MA	3–5	571	0	133	82.2	(71.9, 94.1)	41.7	73.2	148	470
	6–11	887	0.56	161	91.2	(81.9, 101)	45.9	86.1	177	457
	12–18	904	0.66	147	80.4	(68.1, 95.0)	42.6	82.9	156	441
	≥19	3777	0.4	162	86.2	(80.7, 92.0)	41.4	88.5	185	498
BMA	3–5	571	0	26.9	10.6	(9.31, 12.2)	5.58	9.27	17.6	120
	6–11	883	0	13.1	7.39	(6.69, 8.16)	4.08	6.90	11.6	36.7
	12–18	899	0.33	9.09	5.66	(5.00, 6.40)	3.29	6.04	9.71	27.1
	≥19	3777	0.4	11.8	4.63	(4.28, 5.02)	2.42	4.74	8.95	27.9

^a^ 95% confidence interval of the GM; GM, geometric mean; * 40% of samples were below the LOD, so the percentile distribution was reported, but GM was not calculated; LOD, limit of detection; AM, arithmetic mean.

## Data Availability

In this study we used data from the Korean National Environmental Health Survey (KoNEHS) Cycle 3, which was conducted by Ministry of Environment, National Institute of Environmental Research. The data presented are available on request from the corresponding author.

## Supplementary Materials

The following are available online at https://www.mdpi.com/article/10.3390/ijerph19020626/s1, Table S1: Comparison of target chemicals and analytical methods in Cycle 1 (2009–2011), Cycle 2 (2012–2014), and Cycle 3 (2015–2017) of the KoNEHS; Table S2: Concentrations of target chemicals from the national biomonitoring programs of Korea, USA, and Canada. Units for all chemicals are in (μg·L^−1^), except for lead (μg·dL^−1^). For pre- and elementary schoolers, blood lead and mercury were not measured.; Table S3: Concentration distributions of the 26 environmental chemicals for the Korean population KoNEHS Cycle 3 (2015–2017), compared to Cycles 1 and 2. LOD, limit of detection; AM, arithmetic mean; GM, geometric mean. Units are in μg·L^−1^ unless otherwise specified.
