# Supplementary material for: Profile of Environmental Chemicals in the Korean Population—Results of the Korean National Environmental Health Survey (KoNEHS) Cycle 3, 2015–2017
Source: Int J Environ Res Public Health. 2022 Jan 6;19(2):626. doi: 10.3390/ijerph19020626 (PMC8776061; doi:10.3390/ijerph19020626)
Supplement: Supplementary file 1 [file ijerph-19-00626-s001.zip › ijerph-1494110-supplementary.pdf]

Table S1. Comparison of target chemicals and analytical methods in Cycle 1 (2009–2011), Cycle 2 (2012–2014), and Cycle 3 (2015–2017) of the KoNEHS.

Category	Chemicals	Units	Limit of Detection			Analytical instruments
			Cycle 1 (18 items)	*Cycle 2* (21 items)	*Cycle 3* (26 items)	
Whole Blood						
Metals	Lead	μg·dL^{-1}	0.3	0.30	0.30	GF-AAS
	Mercury (total)	μg·L^{-1}	0.2	0.10	0.10	Gold amalgamation direct mercury analyzer
	Manganese	μg·L^{-1}	0.14			GF-AAS
Spot Urine						
Metals	Mercury (total)	μg·L^{-1}	0.2	0.10	0.10	Gold amalgamation direct mercury analyzer
	Cadmium	μg·L^{-1}	0.05	0.050	0.050	GF-AAS
	Arsenic[a]	μg·L^{-1}	0.8			GF-AAS (Hydride generation AAS)
Phthalates metabolites	MEOHP	μg·L^{-1}	0.500	0.26	0.048	UPLC-MS/MS (Cycle 1: HPLC-MS/MS)
	MEHHP	μg·L^{-1}	0.400	0.28	0.056	
	MECPP	μg·L^{-1}		0.34	0.141	
	MnBP	μg·L^{-1}	0.650	0.44	0.04	
	MBzP	μg·L^{-1}		0.27	0.066	
	MCNP	μg·L^{-1}			0.139	
	MCOP	μg·L^{-1}			0.048	
	MCPP	μg·L^{-1}			0.078	
Environmental phenols	Bisphenol A	μg·L^{-1}	0.24	0.15	0.075	UPLC-MS/MS (Cycle 1: HPLC-MS/MS)
	Bisphenol F	μg·L^{-1}			0.084	
	Bisphenol S	μg·L^{-1}			0.019	
	Triclosan	μg·L^{-1}		0.500	0.196	
	Ethyl Paraben	μg·L^{-1}			0.107	
	Methyl Paraben	μg·L^{-1}			0.108	
	Propyl Paraben	μg·L^{-1}			0.082	
Pyrethroid pesticides metabolites	3-Phenoxybenzoic acid	μg·L^{-1}	0.015	0.015	0.015	GC–MS

Tobacco	Cotinine	$\mu g\cdot L^{-1}$	0.3	0.3	0.3	GC–MS
PAHs metabolites	1-Hydroxypyrene	$\mu g\cdot L^{-1}$	0.015	0.015	0.015	GC–MS
	2-Hydroxynaphthalene	$\mu g\cdot L^{-1}$	0.05	0.05	0.05	
	1-Hydroxyphenanthrene	$\mu g\cdot L^{-1}$		0.040	0.040	
	2-Hydroxyfluorene	$\mu g\cdot L^{-1}$		0.04	0.04	
VOC metabolites	Hippuric acid	$g\cdot L^{-1}$	0.00002	0.000015		UPLC-MS (Cycle1: HPLC-MS)
	t,t-Muconic acid	$\mu g\cdot L^{-1}$	3	2.3	2.3	HPLC-MS/MS
	N-Acetyl-S-(benzyl)-L-cysteine	$\mu g\cdot L^{-1}$			0.197	
	Mandelic acid	$mg\cdot L^{-1}$	0.005	0.004		
	Phenylglyoxylic acid	$mg\cdot L^{-1}$	0.006	0.004		
	2-Methylhippuric acid<br>3-Methylhippuric acid<br>4-Methylhippuric acid	$mg\cdot L^{-1}$	0.004<br>0.0038<br>0.003	0.003<br>0.004<br>0.003		

[a]Arsenic = As(III) + As(V) + MMA + DMA

Table S2. Concentrations of target chemicals from the national biomonitoring programs of Korea, USA, and Canada. Units for all chemicals are in ($\mu g\cdot L^{-1}$), except for lead ($\mu g\cdot dL^{-1}$). For pre- and elementary schoolers, blood lead and mercury were not measured.

**National biomonitoring program**	**KoNEHS Cycle 3**				**NHANES** [b] (CDC, 2019)				**CHMS** [c] (Health Canada, 2020)			
Survey years	*2015–2017*				*2015–2016*				*2016–2017*			
Subject age	3–5	6–11	12–18	≥ 19	3–5	6–11	7–19	≥ 20	3–5	6–11	12–19	20–79
Metals												
Lead ($\mu g\cdot dL^{-1}$)[d]			0.802	1.60			0.467	0.920			0.48	F
Mercury (total)[d]			1.37	2.75			0.395	0.810			0.33	0.72
Mercury (total)	0.422	0.394	0.413	0.355		0.241	0.257	*				
Cadmium	0.108	0.232	0.289	0.359		0.057	0.055	0.174	0.22	0.24	0.26	0.22
Phthalates metabolites												
MEOHP	25.5	19.2	9.24	9.88	5.8	5.97	3.75	3.29	8.5	7	4	3.1
MEHHP	34.6	28.8	13.6	13.2	8.65	8.81	5.81	5.29	12	9.7	5.9	4.7

MECPP		45.3	44.5	28.4	23.2	14.9	14.6	9.38	8.12	15	13	6.9	5.5
MnBP		47.2	43.2	36.9	22.3	10.9	14.4	11.6	9.18	20	20	16	11
MBzP		3.12	2.80	2.78	1.99	8.27	10.7	6.05	3.80	7.7 [e]	10	5.3	3.3
MCNP		0.491	0.533	0.452	0.441	2.07	2.26	2.18	1.63				0.63
MCOP		1.62	2.24	1.71	1.07	9.05	11.1	10.3	7.72				0.92
MCPP		1.80	1.56	1.48	1.13	1.72	1.79	1.31	1.06	1.3 [f]	1.3	0.88	0.66
Environmental phenols													
Bisphenol A		2.41	1.70	1.39	1.18		1.43	1.28	1.26	0.94	0.97	0.96	0.78
Bisphenol F					*		0.421	0.567	0.541				
Bisphenol S				0.053	*		0.351	0.4	0.441				
Triclosan		0.513	0.452	0.420	*				10.3	9.5	11	13	*
Ethyl Paraben		14.2	11.4	19.1	30.9				*	*	*	*	*
Methyl Paraben		46.3	28.9	26.1	35.2		28.6	40.5	52.2	9.9 [e]	7.5	9.5	14
Propyl Paraben		4.36	1.83	3.19	3.07				6.27	1.2 [e]	0.96	1.4	2.1 [e]
Pyrethroid pesticides metabolites													
3-Phenoxybenzoic acid		1.08	1.36	1.02	0.965		0.549	0.403		0.4	0.47	0.45	0.55
Environmental tobacco smoke metabolites													
Cotinine	Total	1.05	1.20	3.04	5.59					*	*	F	*
	Non-smokers				1.87					*	*	*	*
	Smokers				524					X [f]	X [f]	620	850
PAHs metabolites													
1-Hydroxypyrene			0.108	0.163	0.130		0.131	0.16	0.128	0.093	0.099	0.11	0.095
2-Hydroxynaphthalene		3.37	2.67	3.05	2.63		3.03	5.09	4.24	3	3.8	4.7	4.7
1-Hydroxyphenanthrene		0.080	*	0.127	0.117		0.068	0.093	0.096	0.1	0.13	0.15	0.16
2-Hydroxyfluorene		0.495	0.209	0.263	0.321		0.119	0.172	0.190	0.16	0.21	0.24	0.3

VOCs metabolites												
trans,trans-Muconic acid	82.2	91.2	80.4	86.2					73	75	74	65
N-Acetyl-S-(benzyl)-L-cysteine	10.6	7.39	5.66	4.63		5.9	5.96	6.25				

[b] Cadmium data in adolescents from 2009–2010; Mercury data in adolescents from 2011–2012; Environmental phenols, PAH and VOC metabolites data from 2013–2014; All others from 2015–2016

[c] Cadmium data in children and adolescents from 2009–2011; Triclosan data in children from 2012–2013; Triclosan, PAH metabolites, and t,t-muconic acid data from 2014–2015; All others from 2016–2017

[d] The matrix of lead and mercury are blood, and those for other chemicals are urine

[e] Use data with caution (This is the original author's information.)

[f] X - Suppressed to meet the confidentiality requirements of the Statistics Act

* 40% of samples were below the LOD; thus, the percentile distribution is reported, but GM was not calculated

Table S3. Concentration distributions of the 26 environmental chemicals for the Korean population KoNEHS Cycle 3 (2015–2017), compared to Cycles 1 and 2. LOD, limit of detection; AM, arithmetic mean; GM, geometric mean. Units are in μg·L^{-1} unless otherwise specified.

**Chemicals**		**Gender**	**Cycle 3**									**Cycle 2**		**Cycle 1**	
			N	*%<LOD*	*AM*	*GM*	*(95% CI)*	*P25*	*P50*	*P75*	*P95*	*N*	*GM*	*N*	*GM*
Whole Blood															
Metals															
Lead (μg·dL^{-1})	12–18	Male	430	5.12	0.991	0.927	(0.867, 0.991)	0.736	0.979	1.21	1.63				
		Female	482	0.62	0.762	0.682	(0.629, 0.740)	0.509	0.682	0.927	1.41				
	≥ 19	Total										6455	1.94	6299	1.77
		Male	1640	0.0	2.08	1.87	(1.81, 1.93)	1.39	1.86	2.48	3.78	2766	2.28	2924	2.16
		Female	2107	0.2	1.51	1.37	(1.34, 1.41)	1.04	1.37	1.84	2.74	3689	1.66	3375	1.46
Mercury (total)	12–18	Male	430	0	1.65	1.44	(1.32, 1.58)	1.13	1.41	1.89	3.07				
		Female	482	0	1.46	1.29	(1.19, 1.39)	0.915	1.29	1.79	2.79				
	≥ 19	Total										6457	3.11	6298	3.08
		Male	1640	0.0	4.22	3.29	(3.09, 3.51)	2.08	3.32	5.18	9.93	2768	3.70	2924	3.65
		Female	2105	0.0	2.78	2.30	(2.20, 2.41)	1.56	2.25	3.36	6.31	3689	2.63	3374	2.62
Spot Urine															
Metals															
Mercury (total)	3–5	Male	285	2.81	0.596	0.438	(0.380, 0.504)	0.280	0.410	0.702	1.28				
		Female	286	1.40	0.532	0.406	(0.356, 0.463)	0.250	0.390	0.633	1.29				
	6–11	Male	452	1.77	0.468	0.394	(0.371, 0.418)	0.280	0.373	0.541	1.03				
		Female	435	0.63	0.70 2	0.393	(0.357, 0.433)	0.250	0.373	0.577	1.20				

	12–18	Male	429	4.43	0.530	0.412	(0.366, 0.464)	0.255	0.421	0.651	1.25				
		Female	477	2.31	0.630	0.415	(0.357, 0.483)	0.237	0.421	0.702	1.53				
		Total										6470	0.382	6297	0.534
	≥19	Male	1647	4.6	0.568	0.400	(0.372, 0.431)	0.223	0.384	0.690	1.50	2769	0.407	2925	0.563
		Female	2133	7.3	0.470	0.315	(0.296, 0.335)	0.178	0.298	0.517	1.36	3701	0.359	3372	0.507
Cadmium	3–5	Male	285	20.0	0.165	0.111	(0.087, 0.142)	0.057	0.099	0.208	0.446				
		Female	286	17.5	0.156	0.105	(0.084, 0.131)	0.056	0.089	0.201	0.414				
	6–11	Male	452	2.21	0.297	0.234	(0.211, 0.258)	0.145	0.234	0.357	0.730				
		Female	435	1.47	0.299	0.231	(0.208, 0.257)	0.148	0.227	0.351	0.720				
	12–18	Male	429	3.03	0.388	0.298	(0.262, 0.339)	0.184	0.312	0.460	0.889				
		Female	477	1.05	0.364	0.279	(0.247, 0.315)	0.180	0.270	0.438	1.04				
		Total										6469	0.376	6296	0.580
	≥ 19	Male	1647	6.2	0.572	0.356	(0.321, 0.394)	0.194	0.416	0.770	1.56	2769	0.387	2925	0.554
		Female	2134	7.2	0.658	0.362	(0.324, 0.405)	0.177	0.429	0.869	1.95	3700	0.366	3371	0.605
Phthalates metabolites															
MEOHP	3–5	Male	285	0	35.9	27.4	(24.3, 30.7)	16.1	28.6	45.9	92.3				
		Female	286	0	32.5	23.7	(20.7, 27.2)	14.3	25.0	36.4	75.8				
	6–11	Male	452	0	25.9	19.7	(17.7, 21.9)	12.1	19.7	32.0	68.5				
		Female	433	0	25.6	18.7	(17.0, 20.6)	11.5	19.1	31.0	63.9				
	12–18	Male	429	0.47	13.9	9.62	(7.88, 11.7)	5.24	10.3	17.8	35.5				
		Female	472	0	14.3	8.83	(7.45, 10.5)	4.80	9.99	16.8	40.8				
		Total										6,392	12.1	6,274	15.5
	≥ 19	Male	1648	0.6	18.3	10.3	(9.23, 11.5)	5.51	11.0	20.9	50.5	2,740	12.2	2,915	15.8
		Female	2133	0.4	17.9	9.47	(8.42, 10.7)	4.60	10.3	20.4	51.7	3,652	12.0	3,359	15.2
MEHHP	3–5	Male	285	0	46.8	36.0	(32.1, 40.4)	22.8	39.0	58.8	99.4				
		Female	286	0	45.0	33.1	(29.0, 37.9)	20.1	33.5	56.6	94.0				
	6–11	Male	452	0	37.8	29.7	(26.9, 32.7)	19.7	30.4	47.0	86.0				
		Female	433	0	37.1	27.8	(25.3, 30.6)	17.8	28.6	47.4	83.5				
	12–18	Male	429	0	20.4	14.6	(12.2, 17.4)	9.07	15.8	26.6	53.5				
		Female	472	0.21	19.3	12.7	(11.0, 14.6)	7.49	13.9	21.9	57.6				
		Total										6,406	17.5	6,274	20.6
	≥ 19	Male	1648	0.5	23.6	14.1	(12.7, 15.6)	7.54	14.5	26.7	65.1	2,745	18.4	2,915	21.8
		Female	2133	0.3	22.6	12.3	(11.2, 13.5)	6.10	12.8	24.8	59.8	3,661	16.6	3,359	19.6
MECPP	3–5	Male	285	0	67.1	46.8	(39.3, 55.6)	26.4	46.6	84.0	176				
		Female	286	0	64.6	43.8	(36.4, 52.6)	25.4	46.6	78.3	168				

	6–11	Male	452	0	58.3	44.8	(40.5, 49.6)	27.8	44.1	70.7	144				
		Female	433	0	59.8	44.2	(40.2, 48.5)	26.0	43.1	73.6	144				
	12–18	Male	429	0	38.4	30.4	(26.0, 35.6)	18.9	31.3	50.8	97.2				
		Female	472	0	35.1	26.3	(23.4, 29.5)	16.7	26.6	40.5	84.1				
		Total										6,406	20.1		
	≥ 19	Male	1648	0.0	40.0	24.0	(21.4, 26.8)	12.5	22.5	45.4	127	2,745	20.5		
		Female	2133	0.0	40.5	22.4	(20.0, 25.0)	10.6	20.8	43.2	139	3,661	19.7		
MnBP	3–5	Male	285	0	93.6	46.7	(37.2, 58.6)	34.6	56.1	78.5	140				
		Female	286	1.05	65.4	47.8	(42.0, 54.4)	28.8	50.0	79.1	173				
	6–11	Male	452	0	57.0	45.1	(41.7, 48.8)	29.5	46.7	69.7	125				
		Female	433	0	54.7	41.3	(38.2, 44.6)	26.4	44.2	68.1	125				
	12–18	Male	427	0	64.6	39.6	(31.0, 50.5)	21.0	38.4	85.0	170				
		Female	470	0.21	56.1	34.0	(26.3, 44.0)	16.9	32.7	70.2	163				
		Total										6,358	23.6	6,274	44.7
	≥ 19	Male	1647	1.0	47.2	24.0	(20.4, 28.1)	13.8	26.5	54.6	133	2,723	24.7	2,915	48.2
		Female	2132	1.1	44.0	20.7	(17.8, 24.1)	11.2	24.0	49.3	123	3,635	22.7	3,359	41.5
MBzP	3–5	Male	285	3.86	8.35	3.27	(2.69, 3.97)	1.67	3.17	6.95	22.9				
		Female	286	3.51	7.77	2.98	(2.35, 3.78)	1.53	3.21	6.73	20.9				
	6–11	Male	452	6.19	6.88	2.94	(2.45, 3.52)	1.48	3.22	7.31	22.8				
		Female	433	5.30	6.81	2.67	(2.13, 3.33)	1.22	3.09	6.82	24.9				
	12–18	Male	429	4.43	7.75	3.37	(2.73, 4.18)	1.53	3.62	8.19	26.8				
		Female	472	3.18	5.95	2.22	(1.82, 2.71)	1.00	2.37	5.75	21.2				
		Total										6,252	2.82		
	≥ 19	Male	1648	1.8	4.58	2.23	(1.99, 2.49)	1.08	2.25	4.60	16.0	2,691	3.05		
		Female	2133	2.2	4.22	1.78	(1.59, 1.99)	0.845	1.83	4.12	13.6	3,561	2.60		
MCNP	3–5	Male	285	3.16	0.711	0.506	(0.430, 0.595)	0.312	0.500	0.710	2.14				
		Female	286	6.64	0.626	0.477	(0.409, 0.555)	0.325	0.444	0.658	1.47				
	6–11	Male	452	1.99	0.643	0.541	(0.506, 0.579)	0.417	0.526	0.673	1.49				
		Female	433	2.33	0.679	0.524	(0.487, 0.564)	0.389	0.525	0.674	1.37				
	12–18	Male	429	3.26	0.528	0.459	(0.432, 0.488)	0.332	0.511	0.604	0.963				
		Female	472	2.97	0.543	0.444	(0.403, 0.488)	0.321	0.490	0.583	1.05				
	≥ 19	Male	1648	11.0	0.608	0.440	(0.390, 0.497)	0.228	0.500	0.778	1.56				
		Female	2133	10.1	0.617	0.442	(0.396, 0.494)	0.228	0.502	0.769	1.45				
MCOP	3–5	Male	285	0	2.15	1.61	(1.41, 1.84)	0.946	1.60	2.63	6.63				
		Female	286	0	2.39	1.62	(1.38, 1.90)	0.873	1.44	2.73	8.50				

	6–11	Male	452	0.22	3.06	2.22	(2.05, 2.41)	1.31	2.11	3.70	8.45				
		Female	433	0	3.72	2.26	(2.01, 2.55)	1.25	2.16	3.94	8.02				
	12–18	Male	429	2.33	2.26	1.69	(1.51, 1.89)	1.12	1.74	2.84	5.70				
		Female	472	1.48	2.57	1.73	(1.53, 1.96)	1.03	1.72	3.06	8.69				
	≥ 19	Male	1648	0.8	1.66	1.11	(0.989, 1.24)	0.622	1.13	2.03	4.45				
		Female	2133	0.5	1.82	1.04	(0.938, 1.16)	0.529	1.02	1.88	4.79				
MCPP	3–5	Male	285	0	2.32	1.82	(1.63, 2.03)	1.31	1.74	2.55	5.37				
		Female	286	0.35	2.24	1.78	(1.59, 1.99)	1.21	1.64	2.62	5.00				
	6–11	Male	452	0.66	1.93	1.61	(1.52, 1.72)	1.13	1.49	2.13	4.48				
		Female	433	0.21	2.01	1.51	(1.42, 1.61)	1.02	1.40	2.07	4.38				
	12–18	Male	429	0.47	1.75	1.53	(1.37, 1.71)	1.08	1.43	2.17	3.60				
		Female	472	0	1.75	1.43	(1.28, 1.60)	0.969	1.28	2.02	4.20				
	≥ 19	Male	1648	1.0	1.59	1.13	(1.01, 1.26)	0.674	0.997	2.10	3.79				
		Female	2133	0.4	1.68	1.13	(1.02, 1.25)	0.671	0.997	1.92	3.88				
Environmental phenols															
Bisphenol A	3–5	Male	285	0.35	3.81	2.24	(1.83, 2.75)	1.41	2.50	4.39	10.4				
		Female	286	2.10	4.87	2.60	(2.16, 3.12)	1.47	2.66	4.33	11.5				
	6–11	Male	452	4.20	3.24	1.71	(1.45, 2.03)	0.825	2.09	3.69	10.1				
		Female	435	3.37	3.21	1.69	(1.45, 1.97)	0.894	1.90	3.48	10.2				
	12–18	Male	429	3.73	2.78	1.48	(1.21, 1.81)	0.829	1.47	2.82	10.4				
		Female	475	2.53	2.50	1.30	(1.10, 1.54)	0.716	1.40	2.91	9.06				
	≥ 19	Total										6,263	1.09	6,266	0.748
		Male	1647	1.8	2.84	1.34	(1.18, 1.53)	0.578	1.52	3.21	8.56	2,685	1.14	2,911	0.811
		Female	2133	2.3	2.19	1.04	(0.93, 1.17)	0.465	1.14	2.43	7.15	3,578	1.05	3,355	0.691
Bisphenol F	3–5	Male	285	73.0	*	*	*	< LOD	< LOD	0.101	0.898				
		Female	286	66.8	*	*	*	< LOD	< LOD	< LOD	0.508				
	6–11	Male	451	64.1	*	*	*	< LOD	< LOD	0.135	1.20				
		Female	433	61.1	*	*	*	< LOD	< LOD	0.13	1.16				
	12–18	Male	429	62.5	*	*	*	< LOD	< LOD	0.183	1.64				
		Female	471	50.1	*	*	*	< LOD	< LOD	0.134	1.37				
	≥ 19	Male	1647	58.7	*	*	*	< LOD	< LOD	0.165	1.48				
		Female	2130	59.7	*	*	*	< LOD	< LOD	0.126	1.08				
Bisphenol S	3–5	Male	285	48.4	*	*	*	< LOD	0.020	0.046	0.163				
		Female	286	46.5	*	*	*	< LOD	0.020	0.045	0.225				
	6–11	Male	451	47.2	*	*	*	< LOD	0.027	0.074	0.580				

		Female	433	41.2	*	*	*	< LOD	0.020	0.056	0.446			
	12–18	Male	429	47.8	0.243	0.056	(0.044, 0.072)	< LOD	0.039	0.133	0.970			
		Female	471	29.5	0.348	0.050	(0.036, 0.069)	< LOD	0.032	0.107	1.23			
	≥ 19	Male	1646	47.7	*	*	*	< LOD	0.022	0.059	0.289			
		Female	2130	44.2	*	*	*	< LOD	0.023	0.056	0.284			
Triclosan	3–5	Male	285	22.1	1.52	0.499	(0.412, 0.605)	0.209	0.387	0.931	4.60			
		Female	286	21.7	1.63	0.529	(0.436, 0.641)	0.204	0.404	1.02	4.86			
	6–11	Male	452	29.6	2.45	0.419	(0.364, 0.483)	< LOD	0.303	0.769	4.47			
		Female	435	32.6	4.66	0.491	(0.420, 0.573)	< LOD	0.345	0.919	7.07			
	12–18	Male	429	42.2	4.41	0.412	(0.354, 0.478)	< LOD	0.332	0.708	2.92			
		Female	475	29.1	3.46	0.430	(0.359, 0.516)	< LOD	0.304	0.716	8.15			
	≥ 19	Total										6,288	*	
		Male	1647	52.6	*	*	*	< LOD	< LOD	0.498	5.01	2,692	*	
		Female	2133	49.6	*	*	*	< LOD	0.202	0.572	5.79	3,596	*	
Ethyl paraben	3–5	Male	285	1.40	122	17.7	(12.1, 25.9)	3.75	24.2	78.8	585			
		Female	286	0.70	89.2	11.2	(7.61, 16.5)	2.29	13.3	55.3	354			
	6–11	Male	451	1.33	140	13.2	(9.75, 18.0)	2.57	12.2	79.9	568			
		Female	433	0.85	101	9.78	(6.90, 13.9)	1.63	8.59	63.6	484			
	12–18	Male	429	0.93	89.6	21.9	(14.4, 33.3)	6.02	25.0	85.9	342			
		Female	471	0	81.1	16.4	(11.9, 22.5)	4.28	14.9	50.0	361			
	≥ 19	Male	1647	0.7	169	36.1	(30.7, 42.5)	10.2	42.3	142	720			
		Female	2132	1.2	147	26.5	(22.4, 31.2)	6.09	30.4	132	668			
Methyl paraben	3–5	Male	285	0	820	55.7	(41.8, 74.3)	10.8	34.3	154	6121			
		Female	286	0	461	38.0	(28.0, 51.5)	7.87	20.2	138	3125			
	6–11	Male	451	0	220	29.6	(24.0, 36.5)	7.14	18.1	90.8	1051			
		Female	433	0	154	28.1	(22.6, 35.0)	7.20	20.1	90.0	563			
	12–18	Male	429	0	71.6	18.6	(14.9, 23.3)	6.47	11.7	55.0	283			
		Female	471	0	147	38.3	(30.2, 48.6)	8.52	35.4	176	577			
	≥ 19	Male	1647	0.0	109	27.3	(24.4, 30.5)	7.48	22.7	104	419			
		Female	2132	0.0	147	45.1	(40.0, 51.0)	14.0	49.1	148	566			
Propyl paraben	3–5	Male	285	0.70	224	4.92	(3.51, 6.90)	0.682	2.82	23.8	1704			
		Female	286	0.70	76.7	3.84	(2.60, 5.68)	0.640	2.30	14.4	374			
	6–11	Male	451	5.76	29.9	1.74	(1.39, 2.19)	0.331	1.29	6.06	100			
		Female	433	4.67	31.5	1.94	(1.49, 2.52)	0.370	1.43	8.37	106			
	12–18	Male	429	2.33	17.5	2.07	(1.57, 2.73)	0.638	1.68	4.68	92.3			

		Female	471	1.91	62.9	5.20	(3.83, 7.05)	1.03	3.46	27.0	345				
	≥ 19	Male	1647	4.8	41.1	1.93	(1.65, 2.26)	0.351	1.24	8.18	156				
		Female	2131	3.3	60.8	4.87	(4.20, 5.65)	0.703	4.31	31.8	270				
Pyrethroid pesticides metabolites															
3-Phenoxy benzoic acid	3–5	Male	285	0.70	2.32	1.02	(0.817, 1.28)	0.488	0.909	1.81	10.2				
		Female	285	0	2.16	1.14	(0.904, 1.43)	0.604	1.02	1.88	8.65				
	6–11	Male	452	1.11	2.54	1.42	(1.25, 1.61)	0.783	1.35	2.56	7.28				
		Female	435	0.21	2.24	1.30	(1.13, 1.50)	0.717	1.23	2.30	7.54				
	12–18	Male	429	2.80	1.93	1.19	(1.00, 1.42)	0.658	1.21	2.34	4.64				
		Female	475	0.63	2.15	0.860	(0.651, 1.14)	0.464	0.988	1.91	6.48				
		Total										6,402	1.41	6,232	1.47
	≥ 19	Male	1643	0.9	1.78	0.956	(0.862, 1.06)	0.498	1.01	2.03	6.09	2,749	1.41	2,893	1.47
		Female	2129	0.9	1.86	0.974	(0.894, 1.06)	0.500	1.05	2.08	6.08	3,653	1.41	3,339	1.48
Environmental tobacco smoke metabolites															
Cotinine	3–5	Male	285	21.1	1.69	1.06	(0.911, 1.22)	0.453	1.21	2.20	4.93				
		Female	286	19.9	1.69	1.04	(0.870, 1.24)	0.442	1.18	2.34	4.73				
	6–11	Male	452	20.1	2.23	1.27	(1.08, 1.48)	0.575	1.53	2.83	6.51				
		Female	435	16.0	2.00	1.14	(0.972, 1.34)	0.384	1.42	2.79	5.28				
	12–18	Male	429	10.3	53.1	4.16	(3.29, 5.27)	1.69	3.45	5.76	309				
		Female	475	4.42	16.9	2.14	(1.69, 2.70)	1.10	2.27	3.67	12.5				
		Total										6,409	5.47	6,268	11.3
	≥ 19	Male	1647	3.4	418	15.1	(12.0, 18.9)	1.08	3.10	670	2000	2,746	19.2	2,912	42.2
		Female	2137	9.3	65.2	2.10	(1.81, 2.44)	0.695	1.30	3.38	417	3,663	1.62	3,356	3.2
PAHs metabolites															
1-Hydroxy pyrene	3–5	Male	278	46.7	*	*	*	< LOD	0.088	0.340	1.13				
		Female	276	48.6	*	*	*	< LOD	0.075	0.363	0.93				
	6–11	Male	441	31.1	0.309	0.100	(0.078, 0.127)	< LOD	0.198	0.438	0.963				
		Female	423	35.1	0.329	0.117	(0.097, 0.141)	< LOD	0.236	0.442	1.05				
	12–18	Male	415	40.2	0.621	0.194	(0.133, 0.283)	< LOD	0.406	0.824	1.81				
		Female	452	23.5	0.603	0.133	(0.086, 0.204)	< LOD	0.266	0.757	2.68				
		Total										6,418	0.151	6,288	0.110
	≥ 19	Male	1637	23.5	0.404	0.158	(0.141, 0.176)	0.071	0.243	0.500	1.39	2,745	0.177	2,918	0.138
		Female	2117	32.2	0.323	0.107	(0.095, 0.120)	< LOD	0.168	0.363	1.07	3,673	0.129	3,370	0.088
2-Hydroxy naphthalene	3–5	Male	278	0.72	7.03	3.68	(3.03, 4.48)	1.80	3.83	7.95	24.5				
		Female	276	1.81	5.52	3.06	(2.57, 3.64)	1.46	2.97	5.92	18.6				

	6–11	Male	441	4.76	5.79	3.05	(2.74, 3.40)	1.64	3.03	5.44	19.7				
		Female	423	2.65	5.46	2.31	(1.91, 2.81)	1.31	2.41	4.91	18.1				
	12–18	Male	414	3.62	5.51	3.05	(2.54, 3.68)	1.70	3.06	6.24	18.5				
		Female	452	1.99	7.19	3.04	(2.45, 3.76)	1.65	3.23	6.84	23.8				
		Total										6,410	2.22	6,286	2.99
	≥ 19	Male	1637	0.7	6.85	3.34	(3.08, 3.61)	1.46	3.01	8.83	23.9	2,750	3.02	2,918	4.35
		Female	2117	1.7	4.34	2.08	(1.94, 2.24)	1.08	2.00	4.26	16.1	3,660	1.64	3,368	2.09
1-Hydroxy phenanthrene	3–5	Male	278	34.8	0.148	0.073	(0.062, 0.087)	< LOD	0.044	0.179	0.484				
		Female	276	42.0	0.165	0.088	(0.073, 0.105)	< LOD	0.074	0.214	0.579				
	6–11	Male	441	40.8	*	*	*	< LOD	0.161	0.361	0.954				
		Female	423	40.0	*	*	*	< LOD	0.171	0.359	0.884				
	12–18	Male	415	43.5	0.274	0.134	(0.112, 0.161)	< LOD	0.169	0.389	0.814				
		Female	451	33.4	0.254	0.119	(0.094, 0.151)	< LOD	0.149	0.360	0.823				
		Total										6,413	0.095		
	≥ 19	Male	1637	29.9	0.314	0.129	(0.117, 0.141)	< LOD	0.154	0.344	0.808	2,746	0.107		
		Female	2114	34.4	0.224	0.106	(0.098, 0.115)	< LOD	0.115	0.262	0.734	3,667	0.085		
2-Hydroxy fluorene	3–5	Male	278	20.5	2.01	0.526	(0.347, 0.799)	0.163	0.632	2.05	8.78				
		Female	271	18.8	2.36	0.464	(0.281, 0.767)	0.086	0.557	1.89	11.0				
	6–11	Male	441	20.6	0.398	0.229	(0.189, 0.277)	0.111	0.325	0.576	1.06				
		Female	423	18.1	0.359	0.191	(0.160, 0.227)	0.062	0.251	0.452	1.13				
	12–18	Male	415	19.0	0.547	0.297	(0.244, 0.361)	0.153	0.363	0.727	1.53				
		Female	452	13.1	0.413	0.228	(0.188, 0.277)	0.111	0.263	0.577	1.31				
		Total										6,397	0.266		
	≥ 19	Male	1637	10.3	0.930	0.433	(0.389, 0.483)	0.212	0.507	1.07	3.44	2,735	0.398		
		Female	2117	17.1	0.467	0.239	(0.217, 0.263)	0.110	0.296	0.589	1.48	3,662	0.180		
VOCs metabolites															
Muconic acid	3–5	Male	285	0	141	88.8	(77.5, 102)	46.0	79.5	149	462				
		Female	286	0	124	75.8	(63.7, 90.2)	38.5	65.6	142	468				
	6–11	Male	452	0.88	167	96.0	(84.6, 109)	47.3	89.5	180	467				
		Female	435	0.21	154	86.2	(75.1, 99.1)	44.5	81.1	175	450				
	12–18	Male	429	1.17	148	86.0	(69.0, 107)	43.6	85.6	163	444				
		Female	475	0.21	146	74.6	(60.7, 91.8)	39.4	79.5	151	440				
		Total										6,376	58.8	6,246	40.8
	≥ 19	Male	1645	0.2	176	102	(96.2, 109)	52.7	107	205	520	2,736	68.8	2,900	49.9
		Female	2132	0.3	148	72.6	(66.3, 79.6)	32.6	73.0	160	480	3,640	50.4	3,346	33.7

N-Acetyl-S-(benzyl)-L-cysteine	3–5	Male	285	0	32.6	11.3	(9.56, 13.4)	5.62	9.27	17.1	126
		Female	286	0	21.0	9.98	(8.39, 11.9)	5.54	9.18	17.7	73.9
	6–11	Male	451	0	10.1	6.71	(6.04, 7.46)	3.75	6.36	10.8	28.1
		Female	432	0	16.3	8.19	(7.13, 9.41)	4.56	7.47	12.9	49.4
	12–18	Male	429	0.47	9.71	5.82	(4.98, 6.81)	3.34	5.92	9.98	28.0
		Female	470	0.21	8.39	5.48	(4.76, 6.30)	3.17	6.19	9.55	25.6
	≥ 19	Male	1646	0.5	9.01	4.65	(4.26, 5.07)	2.49	4.80	8.89	25.5
		Female	2131	0.3	14.5	4.62	(4.21, 5.07)	2.28	4.65	8.99	31.1

*40% of samples were below the LOD; thus, the percentile distribution is reported, but GM was not calculated
